# Sequencing directly from antigen-detection rapid diagnostic tests in Belgium, 2022: a gamechanger in genomic surveillance?

**DOI:** 10.2807/1560-7917.ES.2023.28.9.2200618

**Published:** 2023-03-02

**Authors:** Annabel Rector, Mandy Bloemen, Gilberte Schiettekatte, Piet Maes, Marc Van Ranst, Elke Wollants

**Affiliations:** 1 KU Leuven, Rega Institute, Department of Microbiology, Immunology and Transplantation, Laboratory of Clinical and Epidemiological Virology, Leuven, Belgium; 2 Center for Medical Analysis, Department of Molecular Biology and Immunology, Herentals, Belgium; 3 University Hospitals Leuven, Department of Laboratory Medicine, National Reference Center for Respiratory Pathogens, Leuven, Belgium

**Keywords:** Rapid antigen test (Ag RDT), SARS-CoV-2, influenza virus, adenovirus, rotavirus, Sanger sequencing, Nanopore whole genome sequencing, genomic surveillance

## Abstract

**Background:**

Lateral flow antigen-detection rapid diagnostic tests (Ag-RDTs) for viral infections constitute a fast, cheap and reliable alternative to nucleic acid amplification tests (NAATs). Whereas leftover material from NAATs can be employed for genomic analysis of positive samples, there is a paucity of information on whether viral genetic characterisation can be achieved from archived Ag-RDTs.

**Aim:**

To evaluate the possibility of retrieving leftover material of several viruses from a range of Ag-RDTs, for molecular genetic analysis.

**Methods:**

Archived Ag-RDTs which had been stored for up to 3 months at room temperature were used to extract viral nucleic acids for subsequent RT-qPCR, Sanger sequencing and Nanopore whole genome sequencing. The effects of brands of Ag-RDT and of various ways to prepare Ag-RDT material were evaluated.

**Results:**

SARS-CoV-2 nucleic acids were successfully extracted and sequenced from nine different brands of Ag-RDTs for SARS-CoV-2, and for five of these, after storage for 3 months at room temperature. The approach also worked for Ag-RDTs for influenza virus (n = 3 brands), as well as for rotavirus and adenovirus 40/41 (n = 1 brand). The buffer of the Ag-RDT had an important influence on viral RNA yield from the test strip and the efficiency of subsequent sequencing.

**Conclusion:**

Our finding that the test strip in Ag-RDTs is suited to preserve viral genomic material, even for several months at room temperature, and therefore can serve as source material for genetic characterisation could help improve global coverage of genomic surveillance for SARS-CoV-2 as well as for other viruses.

**Key public health message**

**What did you want to address in this study?**

Both PCR and rapid antigen tests enable to diagnose viral infection. People’s PCR samples, which test positive can be further used to analyse enclosed viral genetic material. This informs on virus characteristics, like the virus type (e.g. A for influenza virus) or variant (e.g. Omicron for SARS-CoV-2), allowing to monitor viral strains circulating in populations. We investigated if antigen test leftovers could also be used to genetically characterise viruses.

**What have we learnt from this study?**

Viral genetic material, extracted from rapid antigen tests, can be successfully employed for genetic analysis and characterisation, even when the tests have been stored up to 3 months at room temperature. The method works for nine brands of SARS-CoV-2 rapid antigen tests, for all three influenza virus tests, as well as one rotavirus and adenovirus test that we investigated, but with varying effectiveness across brands.

**What are the implications of your findings for public health?**

Since rapid antigen tests can provide good source material for further genetic characterisation of viruses by specialised laboratories, a strategy that uses these cheaper and easier tests instead of PCR does not necessarily hamper surveillance of virus types or variants. This approach could be used to improve the global coverage of genomic surveillance for SARS-CoV-2 as well as other viruses.

## Introduction

Following its emergence in December 2019, the severe acute respiratory syndrome coronavirus 2 (SARS-CoV-2) caused a pandemic with, up to July 2022, over half a billion cases of coronavirus disease (COVID-19) and more than 6 million deaths [1]. This brought on major challenges to healthcare systems and authorities worldwide. The availability of effective and safe vaccines has been without question an important element in the way out of this crisis. Nevertheless, the current COVID-19 vaccines are not sufficient to prevent spread and circulation of the virus, even in highly vaccinated populations [2], and are regretfully not accessible for the entire global population [3]. Therefore, epidemiological surveillance through timely and adequate diagnostic testing for SARS-CoV-2 remains vital to mitigate the virus spread and to guide COVID-19 preventive measures.

Currently, two types of tests are being used in the diagnosis of SARS-CoV-2 infection. Molecular assays based on the detection of viral RNA through a nucleic acid amplification test (NAAT), such as real-time reverse transcription quantitative PCR (RT-qPCR), are highly sensitive and specific, but in most cases require expensive laboratory facilities and trained technicians. This makes them less suited for fast scaling-up of testing capacity. An alternative is the detection of viral antigens through immunodiagnostic techniques such as lateral flow antigen-detection rapid diagnostic tests (Ag-RDTs) that can be read visually, or processed and read by an instrument. These Ag-RDTs can be performed outside the laboratory, provide a fast result (usually 15 min) and can be produced fast, cheaply and in large quantities, allowing for swift upscaling of testing capacity. They can be highly specific, but are generally not as sensitive as molecular tests, making them mostly effective for identifying infected persons shedding higher amounts of virus, who are generally most infectious [4,5]. The sensitivity of Ag-RDTs can however vary depending on the brand of the Ag-RDT used and the virus variant tested. Since Ag-RDTs can (in exceptional cases) be negative for samples with higher viral loads, and transmission can also occur from individuals with lower viral loads, it is possible that infectious cases are missed by these tests [6,7].

Although laboratory-based NAAT is still considered to be the reference standard for SARS-CoV-2 diagnosis, the World Health Organization (WHO) recommends the use of Ag-RDTs as a decentralisable, fast and reliable option, provided that the tests meet the WHO standards for Ag-RDTs (≥ 80% sensitivity and ≥ 97% specificity among symptomatic individuals) [8,9].

The continuous evolution of SARS-CoV-2 has already resulted in the emergence of several variants of interest (VOI) and of concern (VOC) which can be associated with increased transmissibility and/or immune escape [10,11]. Therefore, genomic surveillance to allow early identification, detection, monitoring and reporting of emerging variants can be considered as important as epidemiological surveillance in guiding effective mitigation and containment of the virus. Moreover, virus whole genome sequences can be used to investigate spatiotemporal spread and transmission routes, and can help in the design of diagnostic assays, antivirals and vaccines [12-14].

Genomic surveillance of SARS-CoV-2 can be achieved by complete genome sequencing (the golden standard) or by sequencing of a limited viral genome region to detect mutations that are indicative for specific variants and that lead to changes in the receptor binding domain (RBD) of the spike (S) protein of the virus [15,16]. These techniques require the retrieval of viral genetic material from a patient sample, classically the leftover of a nasopharyngeal sample used for NAAT.

Based on our previous experience with the preservation and transport of viral material on paper strips [17-20], we wanted to test whether the leftover viral material in the cellulose carrier in the test strips used in SARS-CoV-2 Ag-RDTs allows extraction of intact viral nucleic acids and subsequent RT-qPCR, Sanger sequencing and/or whole genome sequencing (WGS).

Several other viruses are also frequently diagnosed with Ag-RDTs, and also in these cases obtaining supplementary information regarding the type or variant through sequencing can be of importance. Genomic surveillance of influenza viruses is being performed by laboratories worldwide within the framework of the WHO’s Global Influenza Surveillance and Response System (GISRS). This surveillance provides essential information regarding effectiveness of vaccines and antiviral drugs against currently circulating influenza viruses, vaccine strain selection and the potential spill-over of animal influenza viruses to humans. The surveillance of circulating genotypes of rotavirus is of crucial importance for detection of novel emerging genotypes and/or antigenic drift of strains that can occur through vaccination and can lead to decreased effectiveness or failure of vaccines. For the cases of hepatitis of unknown aetiology among young children that were under investigation by the European Centre for Disease Prevention and Control (ECDC) at the time of writing [21], a potential association with a (novel variant of) human adenovirus 41 (Adv41) is being considered [22,23]. Typing positive adenovirus samples can therefore be of clinical and epidemiological relevance.

In this study, we evaluate the possibility to retrieve leftover material of several viruses from a range of Ag-RDTs, and after different periods of storage at room temperature, for use in molecular genetic analysis.

## Methods

### Samples

For an optimal result, Ag-RDTs need to be performed with fresh samples containing live virus. In practice, samples are usually directly obtained from individuals, when they are symptomatic or suspected of being infected, with a swab provided by the Ag-RDT supplier followed by almost immediate processing with the test. Because, for the current study, it was not feasible to perform all Ag-RDTs in this way, we used anonymised stored nasopharyngeal samples from hospitalised patients as a proxy. For use on an Ag-RDT, such samples need to be stored in phosphate buffered saline (PBS) and not in a virus-inactivating medium that denatures proteins, such as DNA/RNA Shield (Zymo Research, Irvine, California, United States) or InActiv Blue (InActiv Blue bv, Beernem, Belgium), since Ag-RDTs detect intact viral proteins.

In this proof-of-concept study, we used 11 unique samples in PBS (leftovers from NAAT testing with quantification cycle (Cq) < 25, which were stored at 4 °C), as well as four archived Ag-RDTs, on which original nasal or faecal samples had been directly analysed.

### Antigen-detection rapid diagnostic tests 

Ag-RDTs used in this study are described as follows, with an assigned short name in parentheses, for thereafter further referencing in this report.

SARS-CoV-2 Ag-RDTs: Roche SARS-CoV-2 Rapid Antigen Test Nasal, Roche Diagnostics, Basel, Switzerland (**Roche nasal**); FlowFlex SARS-CoV-2 Antigen Rapid Test (Self-Testing), Acon Laboratories Inc., San Diego, California, United States (**FlowFlex**); Newgene COVID-19 Antigen Detection Kit – Nasal Swab, Newgene Bioengeneering, Hangzhou, China (**Newgene**); Coris COVID-19 Ag K-SeT, Coris BioConcept, Gembloux, Belgium (**Coris COVID-19**); Alltest nasal swab test, Hangzhou Alltest Biotech Co. Ltd., Hangzhou, China (**Alltest**); Boson Rapid SARS-CoV-2 Antigen Test Card, Xiamen Boson Biotech Co., Ltd., Xiamen, China (**Boson**).

SARS-CoV-2/influenza dual Ag-RDTs: Orient Gene Influenza and COVID-19 Ag Combo Rapid Test Cassette, Zhejiang Orient Gene Biotech Co., Ltd, Zhejiang, China (**Orient**); AMP Rapid Test CoV-2 Ag + Flu A + B, AMEDA Labordiagnostik GmbH, Graz, Austria (**AMP**); Nadal COVID-19 Ag + Influenza A/B plus test, Nal von minden GmbH, Moers, Germany (**Nadal**).

For adenovirus 40/41 and rotavirus dual Ag-RDTs: the Coris GastroVir K-SeT, Coris BioConcept, Gembloux, Belgium (**Coris Gastro**).

Direct testing of patients with the Ag-RDTs was done as described in the test instruction manual (n = 4 samples). For testing of different Ag-RDTs with positive samples of known viral loads in PBS (n = 11 samples), a total of 100 µL of the PBS solution was mixed with the buffer included in the test kit. The Ag-RDT test was further performed as described in the test instructions manual. To assess the effect of the Ag-RDT test buffer on nucleic acid yield, we also performed Ag-RDT tests without using the buffer solution, by transferring 100 µL of PBS solution directly on to the sample window of the test cassette. Ag-RDTs run with PBS without patient sample were used as negative controls.

### Nucleic acid extraction from antigen-detection rapid diagnostic tests strips

Ag-RDT cassettes were opened by removing the cover, the test strip was taken out and different parts of the test strip were excised with scissors as indicated in the Figure.

**Figure:**
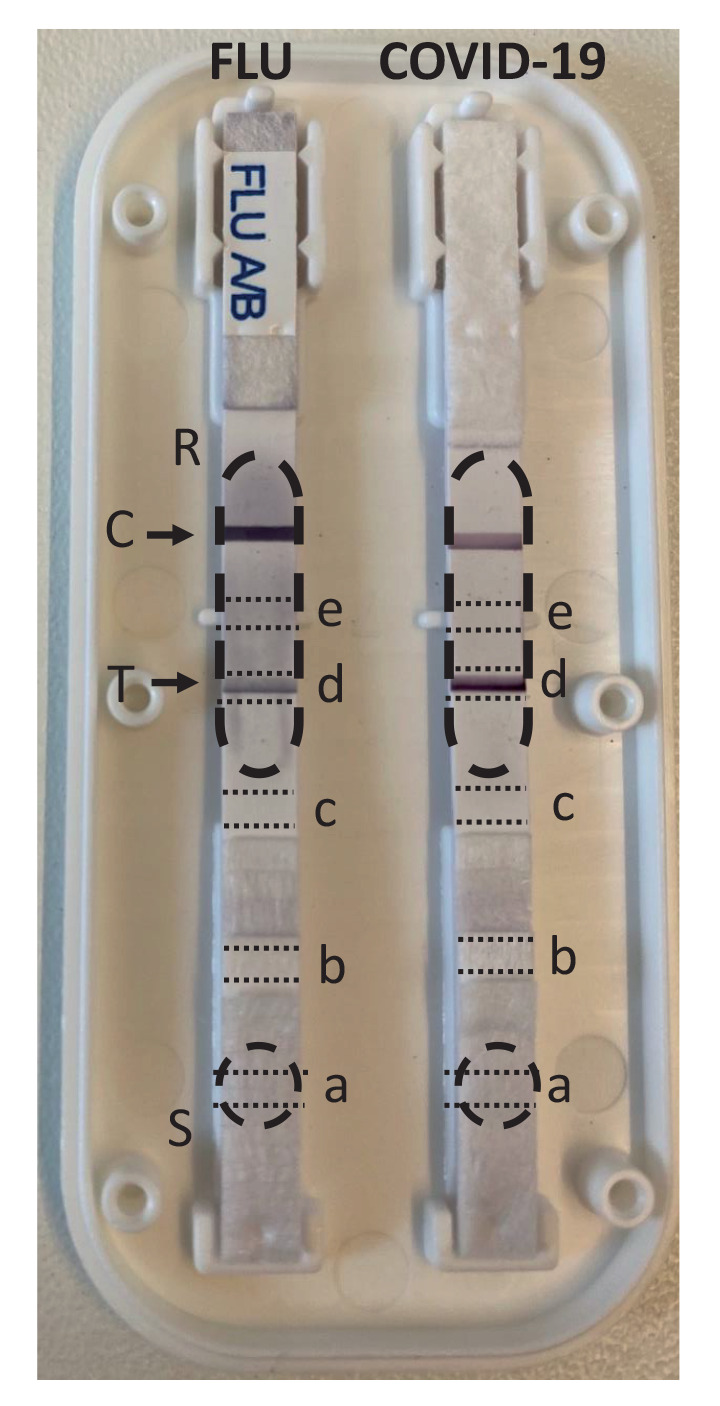
Example of an opened cassette of a lateral flow antigen-detection rapid diagnostic test for influenza and COVID-19^a^, Belgium, 2022

C: control line; COVID-19: coronavirus disease; FLU: influenza; R: result window; S: sample window; SARS-CoV-2: severe acute respiratory syndrome coronavirus 2; T: test line.

^a^ The Orient Gene Influenza and COVID-19 Ag Combo Rapid Test Cassette, Zhejiang Orient Gene Biotech Co., Ltd, Zhejiang, China, is used as an example in this figure.

Thick dashed lines indicate the position of the sample windows (S) and result windows (R). For each respective virus (i.e. SARS-CoV-2 for COVID-19 and influenza virus for influenza), the R window displays a positive test line (T) and a control line (C).

Dotted lines delineate regions (a–e) excised for nucleic acid extraction.

Punch disks were extracted from the strip of unopened Ag-RDT cassettes at the zone of the sample window and at the zone of the positive test line, by using a 2 mm diameter sterile disposable biopsy punch (KAI medical disposable biopsy punch, KAI medical, Japan). The disk or excised part of the Ag-RDT test strip was directly added to 275 µL lysis buffer in a 96 well lysis plate for extraction with the MagMAX Viral/Pathogen II Nucleic Acid Isolation Kit (Applied Biosystems, ThermoFisher Scientific), according to the protocol for KingFisher Flex.

### PCR detection of severe acute respiratory coronavirus 2 and influenza virus 

To detect SARS-CoV-2, an RT-qPCR targeting a region in the N gene was performed on the QuantStudio 7 Flex Real Time PCR system (Applied Biosystems, ThermoFisher). To amplify the N gene region, a reaction mix was made using 5 µL TaqMan Fast Virus 1-step Master Mix (Applied Biosystems), 1.5 µL N2 primer/probe mix from the 2019-nCoV Centers for Disease Control and Prevention Emergency Use Authorization (CDC EUA) kit (IDT) supplemented with 8.5 µL RNase free water to a total volume of 15 µL.

For influenza virus RT-qPCR a reaction mix was made using 5 µL TaqMan Fast Virus 1-step Master Mix (Applied Biosystems) with 0.12 µL forward primer (INFA-3) 5’TCTCATGGAATGGCTAAAGACAAG-3’ (50 µM stock solution) together with 0.2 µL probe (INFA-FP) FAM-5'-TTCACGCTCACCGTGC-3'-MGB (20 µM stock solution) and 0.12 µL reverse primer (INFA-2) 5'-CAAAGCGTCTACGCTGCAGT-3’ (50 µM stock solution) supplemented with 9.56 µL RNase free water to a total volume of 15 µL.

A total of 5 µL of viral RNA was added to the reaction mixes. Thermal cycling conditions were 5 min at 50 °C, 2 min at 95 °C, followed by 45 cycles of 3 s at 95 °C and 30 s at 60 °C. Analysis was done using the QuantStudio Real-Time PCR Software (Applied Biosystems, ThermoFisher).

### PCR and Sanger sequencing for virus typing

PCR and subsequent Sanger sequencing were performed as described in Bloemen et al., 2022 with SARS-CoV-2 primers (S-VOC-F/S-VOC-R) [16]. Additional primers for partial sequencing of influenza virus (AM_FW151/AM_RV397) were described by Schweiger et al., for adenovirus (F-hex1deg/R-hex2deg) by Allard et al. and for rotavirus (F-BEG9/R-END9) by Gouvea et al. [24-26].

### Complete genome sequencing of severe acute respiratory coronavirus 2

Complete genome sequencing of SARS-CoV-2 on RNA extracted from the Ag-RDT was done with the Nanopore technique using the ARTIC protocol as described in Wawina-Bokalanga et al. [27]. Sequences were analysed with Nexclade v 2.0.0 (https://clades.nextstrain.org).

## Results

### Sequencing from archived antigen-detection rapid diagnostic tests

Two different positive SARS-CoV-2 Ag-RDT’s (Roche nasal and Newgene), that had been used according to the test kit instructions to test an infected patient and were kept for documentation purposes, had been stored at room temperature and not shaded from sunlight for 3 months. As a proof of concept, we tested whether residual viral genetic material could be extracted from the test strips. On these strips, the positive test line was still clearly visible, and extraction was done on the positive test strip zone of the test (zone d, indicated in the Figure). For the Roche nasal Ag-RDT, SARS-CoV-2 RNA could still be detected by RT-qPCR, with a Cq value of 27.6. Sanger sequencing on the extracted material allowed to unambiguously determine the virus variant as Omicron (Phylogenetic Assignment of Named Global Outbreak (Pango) lineage designation B.1.1.529) BA.1, based on the partial sequence of the S-gene encoding the RBD (Table: sample code CV-22–2069). We did not have sufficient extracted material left to perform WGS on this initial sample. For the Newgene Ag-RDT (Table: X-22–0205), both Sanger sequencing and WGS were successfully performed and indicated the presence of the Omicron BA.2 SARS-CoV-2 VOC. Likewise, viral material could be retrieved and sequenced from archived Nadal, AMP and Orient SARS-CoV-2/influenza dual Ag-RDTs that had been run with positive nasopharyngeal swab samples in PBS in the frame of a laboratory validation study, and had been stored at room temperature for 3 months thereafter (Table: CM-22–5857; CM-22–1177; BE-22–2238). Sanger and WGS for SARS-CoV-2 were both successful, and although the sequence of sample CM-22–1177 extracted from the Nadal test had only a genome-wide coverage of 75.2%, the variant could be unambiguously determined thanks to the complete coverage of the S gene. For influenza virus, only Sanger sequencing was performed since we do not have a Nanopore sequencing protocol for influenza WGS implemented in our laboratory.

**Table:** Retrieval of viral material from antigen-detection rapid diagnostic tests, Belgium, 2022 (n = 15 samples)

Evaluation of	SAMPLE			Ag-RDT				RESULT			
	Virus	Code	Sample Cq	Name	Use of buffer	∆t^a^	Region^b^	Extract Cq	Sanger sequencing^c^	WGS	WGS genome coverage
**Different archived Ag-RDT**	SARS-CoV-2	**CV-22–2069**	UNK	Roche nasal	YES	3 mths	Zone d	27.6	BA.1	ND	ND
	SARS-CoV-2	**X-22–0205**	UNK	Newgene	YES	3 mths	Zone d	ND	BA.2	BA.2	99.3%
	SARS-CoV-2	CM-22–5857	18.6	AMP	YES	3 mths	Zone d	26.7	BA.2	BA.2	95.2%
				Orient	YES	3 mths	Zone d	25.6	BA.2	BA.2	97.8%
	SARS-CoV-2	CM-22–1177	16.5	Nadal	YES	3 mths	Zone d	20.9	BA.2	BA.2	75.2%
	Influenza A	BE-22–2238	24.6	AMP	YES	3 mths	Zone d	35.4	H3N2	NA	NA
				Orient	YES	3 mths	Zone d	32.2	H3N2	NA	NA
				Nadal	YES	3 mths	Zone d	32.2	H3N2	NA	NA
**Zone of the test strip employed to extract genetic material for analysis **	SARS-CoV-2	CM-22–1177	16.5	AMP	YES	3 mths	Zone a	17.3	BA.2	BA.2	95.9%
							Zone b	22.2	BA.2	ND	ND
							Zone c	19.6	BA.2	ND	ND
							Zone d	21.7	BA.2	BA.2	94.4%
							Zone e	22.6	BA.2	ND	ND
	Influenza A	CM-22–6381	18.5	AMP	YES	3 mths	Zone a	28.6	H3N2	NA	NA
							Zone b	29.9	H3N2	NA	NA
							Zone c	29.3	H3N2	NA	NA
							Zone d	30.6	H3N2	NA	NA
							Zone e	33.3	H3N2	NA	NA
	SARS-CoV-2	CM-22–8672	16.5	Coris COVID-19	YES	24 h	Zone d	28.7	ND	ND	ND
							PD zone d	28.8	ND	ND	ND
**Further usability of virus material after processing a unique positive sample through different Ag-RDTs **	SARS-CoV-2	CM-22–8702	18.5	Alltest	YES	24 h	PD zone a + d	27	BA.2	BA.2	96.5%
				Newgene	YES	24 h	PD zone a + d	21.3	BA.2	BA.2	97.1%
				FlowFlex	YES	24 h	PD zone a + d	20.9	BA.2	BA.2	84.5%
				Boson	YES	24 h	PD zone a + d	26.5	BA.2	BA.2	91.0%
				Coris COVID-19	YES	24 h	PD zone a + d	27.7	BA.2	Failed	Failed
**Influence of the test buffer**	SARS-CoV-2	CM-22–6505	12.6	Roche nasal	YES	72 h	PD zone a + d	32.5	Failed	ND	ND
					NO	72 h	PD zone a + d	19.5	BA.2	BA.2	99.3%
	SARS-CoV-2	CM-22–6516	20.6	Roche nasal	YES	72 h	PD zone a + d	35.8	Failed	ND	ND
					NO	72 h	PD zone a + d	28.8	BA.2	BA.2	86.3%
	SARS-CoV-2	CM-22–3708	18.1	Roche nasal	YES	72 h	PD zone a + d	32.9	Failed	ND	ND
					NO	72 h	PD zone a + d	23.4	BA.4/BA.5	BA.5.1	98.1%
	SARS-CoV-2	CM-22–4853	14.7	Roche nasal	YES	72 h	PD zone a + d	33.3	Failed	ND	ND
					NO	72 h	PD zone a + d	22.0	BA.4/BA.5	BA.4	96.7%
	SARS-CoV-2	CM-22–7973	15.8	Roche nasal	YES	72 h	PD zone a + d	31.2	Failed	ND	ND
					NO	72 h	PD zone a + d	22.9	BA.4/BA.5	BA.5.1	98.4%
**Archived Ag-RDT for other viruses than SARS-CoV-2 and influenza virus**	AdV 40/41	**F14284**	UNK	Coris Gastro	YES	24 h	PD	Positive^d^	AdV 41	NA	NA
	AdV 40/41	**F14298**	UNK	Coris Gastro	YES	24 h	PD	Positive^d^	AdV 41	NA	NA
	Rotavirus	**F14284**	UNK	Coris Gastro	YES	24 h	PD	Positive^d^	G2	NA	NA
	Rotavirus	**F14298**	UNK	Coris Gastro	YES	24 h	PD	Positive^d^	G2	NA	NA

AdV: adenovirus; Ag-RDT: antigen-detection rapid diagnostic test; Cq: quantification cycle; NAAT: nucleic acid amplification test; NA: not available as there was no Nanopore whole genome sequencing protocol implemented in the study laboratory for influenza virus, adenovirus and rotavirus; ND: not done because of insufficient material, because Sanger sequencing was not the point of the particular assay, or because whole genome sequencing was not performed in all cases, given the relatively high cost; PBS: phosphate buffered saline; PD: punch disk extracted from test strip; UNK: unknown; SARS-CoV-2: severe acute respiratory coronavirus 2; WGS: whole genome sequencing.

^a^ Time between adding sample to Ag-RDT and nucleic acid extraction from the test strip.

^b^ Region of test strip excised with scissors (unless indicated as PD) and used for extraction, as indicated in the Figure.

^c^ Results is indicated as virus variant determined by sequencing or ‘failed’ when no sequencing data were obtained.

^d^ For AdV 40/41 and rotavirus, a Cq value was not obtained, but only a positive or negative result, because for those viruses, a regular diagnostic PCR, rather than a quantitative PCR, is used in the study laboratory, followed by polyacrylamide gel electrophoresis and Sanger sequencing for further characterisation of these viruses.

Sample codes in bold were used Ag-RDT test cassettes (with patient samples tested directly on the Ag-RDTs as described in the test instruction manual), other sample codes were stored leftover samples from NAAT in PBS that were subsequently used on an Ag-RDT.

### Evaluation of specimen preparation

The lateral flow rapid Ag tests used in this study all contain a test strip on which the sample is added via the test window. The sample is absorbed onto the sample pad of the test strip, and is transported via capillary flow to the distal end of the strip, flowing over the conjugate pad, test line and control line. To investigate in which area of the test strip the largest amount of viral material from the sample is maintained, and is thus best suited to be used to extract viral material, different zones of the test strip were excised with scissors and used for nucleic acid extraction and RT-qPCR (Figure). A dual antigen test for influenza and COVID-19 (AMP Rapid Test CoV-2 Ag + Flu A + B) that had been run with an influenza virus positive sample (CM-22–6381) and a SARS-CoV-2 positive sample (CM-22–1177), was used for nucleic acid extraction 3 months after the Ag test was run. The viral nucleic acid yield at different positions was determined by RT-qPCR for influenza A virus and SARS-CoV-2 respectively (Table). At all positions that were tested, viral genomic material could be harvested, with the sample pad (zone a) resulting in the highest yield for both SARS-CoV-2 and influenza virus, and the zone distal from the test line (zone e) giving the lowest yield for both viruses. We were able to successfully perform Sanger sequencing on all extracts.

For SARS-CoV-2 sample CM-22–1177, WGS was only performed on zone a and d of the test pad given the high cost.

To evaluate the possibility of performing extraction from Ag-RDTs in a high throughput environment, we also tested the use of a punch disk obtained from the strip at the test line. These punch disks can be retrieved without opening the test cassette. Employing a punch disk vs excision of the test line zone of the strip was compared using a Coris COVID-19 Ag test, and the resulting Cq values after extraction were approximately the same (Table: CM-22–8672). Since the procedure to retrieve the punch from the strip can be standardised and is relatively easy to use, we decided to use punch disks in further experiments.

To evaluate whether viral material is conserved at a comparable degree in different brands of SARS-CoV-2 Ag-RDTs, tests that were commonly used in Belgium at the time of this investigation were run in parallel using the same positive sample (Cq of 18.5; CM-22–8702). This resulted in a clear positive test line on all tests that were analysed. Twenty-four hours after performing the Ag-RDT, punch disks were taken from both the test line zone (zone d) and the sample pad (zone a) of the test strip and the obtained disks were combined for nucleic-acid extraction, followed by RT-qPCR, Sanger sequencing and WGS. All brands of SARS-CoV-2 Ag-RDTs that were tested using this approach allowed detection of SARS-CoV-2 by RT-qPCR, and in all cases variant typing by Sanger sequencing was possible (Table). This indicates that conservation of viral material in a used Ag-RDT did not only occur in one specific brand, and that SARS-CoV-2 Ag-RDTs in general can be used for genomic surveillance. We did, however, notice quite extensive differences in the RNA yield that could be obtained, as indicated by the SARS-CoV-2 Cq values after extraction, which ranged from 20.9 to 27.7. Samples extracted from Alltest, Newgene and Boson resulted in WGS data of good quality whereas the FlowFlex sample was typable by WGS but the genome coverage was 84.5%. WGS failed on the sample extracted from Coris COVID-19 (Cq > 27).

Since the Ag-RDTs use buffers with different compositions, the effect of the buffer on RNA yield was tested. Five different samples were loaded on Ag tests with and without addition of the test kit buffer (Table, test performed on Roche nasal). For the five samples tested, the Cq value of the extract was around 10 cycles higher when buffer was used, which equals approximately a 1,000-fold lower concentration of viral genomic material. When no buffer was used, the results of Sanger sequencing were successful and, in general, data of good quality were obtained by WGS. This indicates that some buffer components are deleterious for the viral genetic material, resulting in lower RNA concentrations and interfering with sequencing. WGS was not performed on samples that failed for Sanger sequencing given the high cost of Nanopore sequencing.

### Material retrieval from antigen-detection rapid diagnostic tests for influenza virus, adenovirus and rotavirus 

An influenza A virus positive sample (nasopharyngeal swab in PBS; BE-22–2238), that had been tested in the routine diagnostic laboratory of the Belgium National Reference Center (NRC) for Respiratory Pathogens in Leuven), had also been run on the influenza test strip of the AMP, Orient and Nadal SARS-CoV-2/influenza virus dual antigen tests. A positive test line for influenza A virus had been obtained with all three Ag-RDTs. Three months after performing the Ag-RDTs, the test line zones were excised with scissors, and viral genetic material was extracted. After extraction, sequences of influenza A virus could be retrieved by Sanger sequencing of part of the M gene for all three Ag-RDTs (Table).

Two faecal samples that had been processed in the NRC on Coris GastroVir K-SeT Ag-RDTs for adenovirus and rotavirus diagnostics were tested for virus material recovery. This Ag-RDT contains only one test strip, but the strip includes two test lines (one for adenovirus and one for rotavirus). Sample F14298 displayed a positive test line for both adeno- and rotavirus on the strip, while sample F14284 displayed a positive test line for rotavirus but not for adenovirus. Punch disks were taken from the sampling pad, the rotavirus test line and the adenovirus test line of the strips. After extraction of virus material, PCR and Sanger sequencing of part of the VP7 gene were performed for rotavirus detection on material from each disk separately. For both samples we detected the presence of rotavirus G2 in extractions of the disks taken at all three sampling sites of the test strip. The adenovirus PCR was also positive for both samples, and sequences of adenovirus 41 were retrieved, again at all three sampling sites of the test strip, also in the sample that did not display a positive test band (Table). WGS was not done on these viruses since we do not have this protocol implemented for Nanopore sequencing in our laboratory.

## Discussion

Whereas RT-qPCR continues to be the gold standard for COVID-19 diagnosis, rapid Ag-RDTs are widely used since they offer the benefit of a very short time to results, are cheaper, and easy to use. A disadvantage of testing through Ag-RDTs so far has been that no leftover sample material is available for genomic characterisation of positive samples. Our finding that the test strips used in several Ag-RDT brands are suited to preserve intact viral nucleic acid material, even for months and when stored at room temperature, and can serve as source for genomic characterisation of the virus, could be a gamechanger in the COVID-19 surveillance strategy.

We demonstrated that a SARS-CoV-2 Ag-RDT that was stored at room temperature up to 3 months could still serve as source material for genomic surveillance. This implies that used Ag test can be easily sent to sequencing facilities, even by regular mail. This provides an easy and cheap way to establish baseline genomic surveillance in countries/regions with limited resources, which would otherwise remain blind spots for surveillance, thus reducing the chance of not detecting novel emerging virus variants. This could therefore mean an important improvement in the global coverage of genomic surveillance.

In this study, we investigated a total of nine different Ag-RDTs for SARS-CoV-2 detection (with or without concurrent influenza virus detection), and were able to successfully perform Sanger sequencing on extracted material from all of these. The difference between the original Cq in the sample and the Cq value after extraction of the test strip largely differed depending on the Ag-RDT used. We tested, for one brand, if the buffer supplied with the Ag-RDT might have an effect in this respect. When samples were directly applied on the Ag-RDT cassette without buffer, the yields of the RT-qPCRs were higher than for samples applied with buffer; samples without buffer thereafter had good sequencing results, both by Sanger and by WGS, suggesting that the buffer from Ag-RDTs may influence the yield of RNA retrieved from the test strip and the subsequent sequencing. We, however, do not recommend using Ag-RDTs without their designated buffers, since this might result in lowered efficiency of viral antigen detection, and in this way could lead to false negative test results. Instead, when further genetic characterisation of a virus is foreseen, an Ag-RDT for which the buffer interferes as little as possible with subsequent sequencing should be chosen.

Nazario-Toole et al. demonstrated recently that WGS of SARS-CoV-2 from excess clinical specimens processed using the Abbott BinaxNOW COVID-19 Ag Card was feasible [28]. Two recent studies also describe that genomic surveillance of SARS-CoV-2 can be performed by sequencing of complete genomes from used rapid antigen tests, with both reporting on the same Ag-RDT brand (the Abbott Panbio COVID-19 Ag Rapid Test Device) [29,30]. However, none of these studies assessed the effect of prolonged room temperature storage of completed Ag-RDTs, nor did they study different brands of SARS-CoV-2 Ag-RDTs, or Ag-RDTs for other viruses.

Our results suggest that this method could be more widely used, as we demonstrated that genomic material of influenza virus, adenovirus and rotavirus can also be retrieved from Ag-RDTs. Especially during the upcoming winter seasons, when an upsurge in COVID-19 is to be expected with concomitant circulation of other respiratory pathogens such as influenza virus, the use of Ag-RDTs that allow combined detection of multiple respiratory pathogens, can prove to be a very cost-efficient testing strategy. The use of these Ag-RDTs could potentially be integrated in genomic surveillance, including the monitoring of novel variants. However, if future genomic surveillance would mainly rely on sequencing from used Ag-RDTs, the risk that novel variants could affect the accuracy of available Ag-RDTs and might therefore remain undetected, should be taken into account.

Limitations of our study are the small number of samples investigated, especially for influenza virus, rotavirus and adenovirus. Studies on larger sample sets, including samples with lower viral loads and taken at different stages of infection, are needed to confirm our results and to determine limits of detection. The effect of the Ag-RDT’s test buffer on RNA yield and subsequent sequencing results was only investigated in one of the Ag-RDTs and should be further explored on a broad range of tests and buffers.

## Conclusion

In this proof-of-concept study, we were able to confirm the usability for 10 commercial Ag-RDTs which are commonly used in Belgium. We plan to further optimise the method for high-throughput processing with samples tested directly on Ag-RDTs. Since it was not possible to test all commercial Ag-RDTs, and tests were now only performed on limited numbers of samples, all with high viral load (Cq < 25), validation experiments on larger numbers of samples with greater variety in Cq range should be performed for each brand before large scale implementation of a certain Ag-RDT in genomic surveillance.
